# Start-to-end simulation of single-particle imaging using ultra-short pulses at the European X-ray Free-Electron Laser

**DOI:** 10.1107/S2052252517009496

**Published:** 2017-09-01

**Authors:** Carsten Fortmann-Grote, Alexey Buzmakov, Zoltan Jurek, Ne-Te Duane Loh, Liubov Samoylova, Robin Santra, Evgeny A. Schneidmiller, Thomas Tschentscher, Sergey Yakubov, Chun Hong Yoon, Michael V. Yurkov, Beata Ziaja-Motyka, Adrian P. Mancuso

**Affiliations:** a European XFEL GmbH, Holzkoppel 4, 22869 Schenefeld, Germany; b FSRC ‘Crystallography and Photonics’, Russian Academy of Sciences, Moscow, Russian Federation; c Center for Free-Electron Laser Science, DESY, Notkestrasse 85, 22607 Hamburg, Germany; d The Hamburg Center for Ultrafast Imaging, Luruper Chaussee 149, 22761 Hamburg, Germany; e Centre for Bio-Imaging Sciences, National University of Singapore, Singapore; f Department of Biological Sciences, National University of Singapore, Singapore; g Department of Physics, National University of Singapore, Singapore; h Department of Physics, University of Hamburg, Jungiusstrasse 9, 20355 Hamburg, Germany; i DESY, Notkestrasse 85, 22607 Hamburg, Germany; j Linac Coherent Light Source, SLAC National Accelerator Laboratory, 2575 Sand Hill Road, Menlo Park CA 94025, USA; k Institute of Nuclear Physics, Polish Academy of Sciences, Radzikowskiego 152, 31-342 Krakow, Poland

**Keywords:** single-particle imaging, X-ray free-electron lasers, simulations, diffraction, scattering

## Abstract

Single-particle imaging with X-ray free-electron lasers (XFELs) has the potential to provide structural information at atomic resolution for non-crystalline biomolecules. This potential exists because ultra-short intense pulses can produce interpretable diffraction data notwithstanding radiation damage. This paper explores the impact of pulse duration on the interpretability of diffraction data using comprehensive and realistic simulations of an imaging experiment at the European X-ray Free-Electron Laser. It is found that the optimal pulse duration for molecules with a few thousand atoms at 5 keV lies between 3 and 9 fs.

## Abstract 2

The optimal XFEL pulse duration for single-particle imaging of small proteins is narrowed down to the 3–9 fs range, using start-to-end simulations of a single-particle imaging experiment at the European XFEL.

## 1. Introduction   

Resolving the atomic structure of biologically relevant macromolecules on length scales of a few ångströms (10^−10^ m) is a key challenge in structural biology. X-ray free-electron lasers (XFELs) are expected to advance this field due to their unprecedented levels of X-ray fluence and peak brightness and, simultaneously, their ultra-short pulse duration from a few up to a few tens of femtoseconds. These intense pulses are capable of probing the sample before radiation damage processes significantly alter and ultimately destroy it (Neutze *et al.*, 2000 ▸) and, due to their extreme intensity, they can compensate for the inherently weak scattering efficiency of a single molecule, such that diffraction patterns with sufficient signal and signal-to-noise levels for the reconstruction of three-dimensional structures can be observed. During a single-particle imaging (SPI) experiment, a large number of two-dimensional diffraction patterns from individual particles (*e.g.* molecules, clusters, or biological specimens like cells or viruses) are recorded. Since the orientation of the sample with respect to the beam and the detector is unknown, the individual patterns must be oriented and merged into a three-dimensional diffraction volume before the three-dimensional electron-density map is reconstructed *via* phase retrieval (Fienup, 1982 ▸).

Electron-density reconstruction of the three-dimensional electron density from experimental SPI data in the soft X-ray regime (Ekeberg *et al.*, 2015 ▸; Seibert *et al.*, 2011 ▸) has so far achieved resolutions in the regime of a few tens of nanometres. Diffraction data at a theoretical resolution of 5.6 Å were measured (Munke *et al.*, 2016 ▸) but not enough patterns were recorded for reconstruction. A comprehensive summary of SPI results from the Linac Coherent Light Source (LCLS) is given by Barty (2016 ▸), along with several references to work on imaging of larger particles (*e.g.* cells) using synchrotrons and two-dimensional imaging. Despite these encouraging results, SPI at a resolution of a few ångströms is still regarded as a severe challenge (Aquila *et al.*, 2015 ▸), in particular with respect to delivering the sample molecules at a high repetition rate (Daurer *et al.*, 2017 ▸) and with a narrow size distribution. A discussion of these and other challenges can be found in the literature [*e.g.* Aquila *et al.* (2015 ▸), Ziaja *et al.* (2015 ▸) and Barty (2016 ▸)].

Among these challenges, a detailed understanding of the radiation damage incurred by the sample, and of the performance of the reconstruction algorithms applied to low signal-to-noise diffraction patterns, has recently received increased attention and is also the focus of the present paper.

### 1.1. Radiation damage   

Electronic radiation damage or ionization begins with the very first photons hitting the sample, producing *K*-shell photoelectrons with a kinetic energy of a few hundred to a few thousand electronvolts (Cryan *et al.*, 2010 ▸; see also Berrah, 2016 ▸). This process is followed by Auger decay (Hau-Riege *et al.*, 2004 ▸; Moribayashi & Kai, 2009 ▸; Lorenz *et al.*, 2012 ▸); Auger lifetimes of the most abundant atoms in biomolecules lie between 4.9 fs (oxygen) and 10.7 fs (carbon) (Ziaja *et al.*, 2015 ▸; Hubbell *et al.*, 1994 ▸). The immediate effect of ionization is a decrease in the number of coherently scattered photons as the elastic scattering cross section scales with the square of the number of bound electrons, whereas ionized electrons predominantly scatter incoherently, thus contributing to the background signal (Slowik *et al.*, 2014 ▸; Gorobtsov *et al.*, 2015 ▸).

Auger electrons from *L* or *M* shells leave the atom with an energy of a few hundred electronvolts, triggering an avalanche of secondary impact ionization (Kai & Moribayashi, 2009*a* ▸,*b* ▸) on time scales of roughly 10–100 fs, creating the strong repulsive forces between ions responsible for Coulomb expansion (Hau-Riege *et al.*, 2004 ▸; Ziaja *et al.*, 2006 ▸). Typical ion velocities in the sample reach of the order 0.1 Å fs^−1^, hence already limiting the resolution to ≃10 Å levels after tens of femtoseconds. The onset of plasma expansion is related to the effect of electrostatic trapping (Hau-Riege *et al.*, 2004 ▸), when the positive charge of the ionized molecule is so high that further ionized electrons can no longer escape from the system, and leads to a drastic increase in the impact ionization rate.

Theoretical treatments of radiation damage fall into two categories: atomistic first-principles simulations (Moribayashi, 2010 ▸; Son *et al.*, 2011 ▸; Lorenz *et al.*, 2012 ▸; Gorobtsov *et al.*, 2015 ▸; Jurek *et al.*, 2016 ▸; Ho & Knight, 2017 ▸) describing the sample dynamics on the level of individual particles including the quantum electrodynamics of electrons in intense X-ray fields, and continuum models operating on distribution functions and densities rather than particles. Continuum models (Hau-Riege *et al.*, 2004 ▸; Ziaja *et al.*, 2006 ▸; Moribayashi, 2008 ▸; Quiney & Nugent, 2010 ▸; Kai *et al.*, 2013 ▸) are numerically less expensive, allowing simulations of large systems on modest computer hardware. However, if kept on the level of single-particle densities, they neglect the correlations between particles. For a treatment of two-particle correlations, see Jurek *et al.* (2012 ▸).

### 1.2. Orientation recovery   

The expand–maximize–compress (EMC) algorithm (Loh & Elser, 2009 ▸) is often used in the SPI community, not least thanks to its user-friendly and open-source implementation (Ayyer *et al.*, 2016 ▸). EMC is an extension of the expectation–maximization technique described by Dempster *et al.* (1977 ▸). Similar reconstruction algorithms that apply Bayesian inference are used in three-dimensional cryoelectron microscopy as well (Scheres *et al.*, 2007 ▸). Generative topographical mapping (GTM; Svensen, 1998 ▸) has also been applied to a partial subspace of a full three-dimensional rotation group (Fung *et al.*, 2009 ▸). A formal comparison of EMC and GTM can be found in the work by Moths & Ourmazd (2011 ▸).

EMC starts from a random initialization of a three-dimensional diffraction volume, the reference model, which is then iteratively updated to maximize overlap with the measured (simulated) two-dimensional patterns until the relative change in voxel intensities stays below a given threshold in two subsequent iterations. The use of a reference model has the advantage that EMC’s complexity is 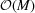, where *M* is the number of recorded diffraction patterns. Alternative methods classify patterns based on their mutual cross correlation (Huldt *et al.*, 2003 ▸; Bortel & Faigel, 2007 ▸), giving 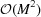 complexity. Patterns of the same class are then averaged to amplify the signal-to-noise ratio and a three-dimensional diffraction pattern is assembled using the ‘common-arc’ method, as described by Huldt *et al.* (2003 ▸) and demonstrated by Bortel & Tegze (2011 ▸). The latter authors have also developed an orientation scheme suitable for large molecules and noisy patterns (Tegze & Bortel, 2012 ▸). A graph-theoretical manifold-embedding technique is described by Giannakis *et al.* (2012 ▸) and applied by Schwander *et al.* (2012 ▸). Quiney & Nugent (2010 ▸) show a way of orienting the measured patterns and directly reconstructing the atomic positions without the need for determining a three-dimensional electron distribution first. This method makes use of the fact that the disturbed electron distribution imprints features of a partially coherent wavefield on the scattered radiation, allowing the treatment of electronic radiation damage and orientation recovery within a unified framework. Multi-tiered iterative phasing (Donatelli *et al.*, 2015 ▸) is a rather novel method that combines the orientation and phasing steps of coherent diffraction imaging into one algorithm.

### 1.3. Scope of this paper   

The robustness and fidelity of orientation and phasing algorithms depends on the signal-to-noise level of the measured diffraction patterns. Previous theoretical predictions (Son *et al.*, 2011 ▸; Gorobtsov *et al.*, 2015 ▸) indicate that the resolution of SPI should increase with decreasing FEL pulse duration at a fixed fluence (number of photons per surface area) of the incoming X-ray pulse; see also the discussions in the articles by Aquila *et al.* (2015 ▸) and Ziaja *et al.* (2015 ▸). In this work, we take a closer look at the question of preferential experimental parameters for SPI, taking into account available machine parameters. In particular, the maximum available X-ray pulse energy in an FEL based on self-amplification of spontaneous emission (SASE) decreases at shorter pulse durations (Schmüser *et al.*, 2009 ▸; Pellegrini *et al.*, 2016 ▸), so these properties cannot be varied independently of each other.

We investigate the impact of pulse duration on simulated diffraction patterns exploiting comprehensive simulations (Yoon *et al.*, 2016 ▸) of an imaging experiment at the European XFEL (Altarelli, 2015 ▸) under realistic conditions. Our simulations track the X-ray fields from their generation in the FEL’s undulator structure through the X-ray optical beamline to the sample interaction point. Subsequently, we model the X-ray interaction with the sample and scattering from it, including time-dependent effects and their eventual registration in the detector. Orientation and phasing (Loh & Elser, 2009 ▸) of the simulated diffraction patterns are also part of the simulation workflow.

Yoon *et al.* (2016 ▸) showed that reducing the pulse duration from 30 to 9 fs markedly improves the speckle contrast in diffraction patterns and the consistency of oriented diffraction volumes, and ultimately the agreement of reconstructed electron densities with crystallographic data. A rather small sample molecule (PDB entry 2nip) was used in that study. These results support the theoretical argument in favour of ultra-short pulses of only a few femtoseconds duration being capable of probing the sample before atomic displacement reaches a level that becomes prohibitive for ångström-level resolution. Here, we study whether we can further improve the signal level and signal-to-noise ratio, and thereby in turn the consistency of oriented diffraction volumes, by reducing the pulse duration to 3 fs, *i.e.* shorter than the Auger lifetime of typical biomolecule constituents. We use the same study molecule and compare our predictions with the earlier results of Yoon *et al.* (2016 ▸).

We employ only one method or algorithm for each simulation step and explore the variation in experimental observables (diffraction patterns and their orientation) as a function of experimental parameters (in particular pulse duration) within this fixed framework. Whether and to what extent our results change if different algorithms are employed is an important open question that will be addressed elsewhere.

## 2. Details of the simulation workflow   

### 2.1. XFEL source and wave propagation to the sample   

The XFEL Photon Pulses Database (XPD, https://in.xfel.eu/xpd/), operated by European XFEL GmbH, provides precomputed pulses at the undulator exit for a large range of accelerator energies, bunch charges, undulator lengths and photon energies at the European XFEL. The database is populated with results from the *FAST* code (Saldin *et al.*, 1999 ▸). Here, we pick 4.96 keV X-ray photons emitted from 12 GeV electrons, the same parameters as used by Yoon *et al.* (2016 ▸). The X-ray pulse durations are 3, 9 and 30 fs, containing approximately 1 × 10^11^, 5 × 10^11^ and 1 × 10^12^ photons per pulse, respectively.

We query 40 pulses from the database to sample the shot-to-shot fluctuations of the temporal structure of SASE pulses. We propagate the X-ray laser pulses through the SASE1 beamline and the focusing optics at the SPB/SFX instrument (Mancuso *et al.*, 2013 ▸; Bean *et al.*, 2016 ▸) using the Fourier optical wave propagation code *WPG* (Chubar *et al.*, 2002 ▸; Samoylova *et al.*, 2016 ▸), which yields the intensity and phase distribution as a function of time at the sample position.

From the propagated pulse data, we convert the time-dependent X-ray intensity into a photon number, which is then used in the subsequent simulation steps. Other pulse properties, such as the curvature of the wavefront, the pointing stability and the related hit statistics, are neglected, *i.e.* we assume that each sample molecule is fully exposed to the brightest part of the X-ray pulse. The fine structure of the source spectrum is also neglected as it only becomes important in the vicinity of an absorption edge or a resonance line of one of the sample elements, which is not the case at our photon energy of 5 keV.

### 2.2. The sample   

Our simulated sample is the two-nitrogenase iron protein (2nip). Diffraction from 2nip for 9 and 30 fs pulse durations at 4.96 keV was simulated by Yoon *et al.* (2016 ▸). We compare these reference data to our new results for diffraction of 3 fs X-ray pulses. All other X-ray pulse parameters (photon energy, active undulator length of 35 m, focusing optics and detector geometry) are the same as used by Yoon *et al.* (2016 ▸). Note that the rather small 2nip (∼7 nm in diameter) is not a typical candidate for SPI experiments at the European XFEL. It is studied here mainly for the pragmatic reason that simulations of much larger particles with our techniques become numerically expensive and a pulse duration scan as presented in this work would not be possible within a reasonable time on our current computing infrastructure.

We ignore the fact that the sample is typically embedded in a solvent (see *e.g.* Wang *et al.*, 2011 ▸). The solvent has two counteracting effects. On the one hand, theory predicted (Hau-Riege *et al.*, 2004 ▸, 2007 ▸; Jurek & Faigel, 2008 ▸) and experiments confirmed (Hau-Riege *et al.*, 2010 ▸) a tampering effect of the solvent layer, mitigating the effect of radiation damage. On the other hand, the solvent layer increases the background scattering, thus reducing the signal-to-noise ratio. Both effects are size dependent and it can be expected that an optimal solvent layer thickness exists, which mitigates radiation damage as much as possible while keeping the diffraction background tolerable. Simulations that investigate this aspect are in progress.

### 2.3. Radiation damage and diffraction   

Our study aims to assess the interpretability of diffraction patterns and the potential for reconstruction of the molecular structure at atomic resolution, hence we employ a molecular dynamics (MD) scheme to describe the sample and its interaction with the X-ray pulse. This provides the required atomistic spatial accuracy. We use the code package *XRAYPAC* (Centre for Free Electron Laser Science Theory Division, 2016 ▸), which combines the MD code *XMDYN* (Murphy *et al.*, 2014 ▸; Jurek *et al.*, 2016 ▸) for electron and ion real-space dynamics with a Monte Carlo code modelling inner-shell electronic transitions and subsequent inelastic electron scattering and recombination events. Rates and cross sections are read from the tabulated output of the *ab initio* electronic structure code *XATOM* (Son *et al.*, 2011 ▸), which is also part of *XRAYPAC*. Other implementations of this atomistic approach to radiation damage are presented by Moribayashi (2010 ▸) and, more recently, by Ho & Knight (2017 ▸). *XMDYN* and *XATOM* have been successfully used to interpret spectroscopy experiments (Rudek *et al.*, 2012 ▸; Fukuzawa *et al.*, 2013 ▸; Murphy *et al.*, 2014 ▸; Tachibana *et al.*, 2015 ▸). The rate equation approach underlying *XATOM* has also been applied in investigations of radiation damage in biological samples (Lorenz *et al.*, 2012 ▸; Gorobtsov *et al.*, 2015 ▸) that completely neglected atomic displacement for pulse durations shorter than 40 fs, citing self-termination of diffraction on these time scales observed in nanocrystallographic diffraction measurements by Barty *et al.* (2011 ▸).

It should be noted that there are important differences between serial femtosecond crystallography (SFX) and SPI, which make this assumption questionable. In SFX (Chapman, 2015 ▸; Schlichting, 2015 ▸) the gating effect applies, *i.e.* as soon as the crystalline lattice is destroyed, Bragg diffraction, the dominant contribution to the overall signal, is ‘switched off’. Only incoherent scattering remains, enhancing the background, but always at levels which are small compared with the already accumulated Bragg signal. In SPI, there is no lattice to start with and such self-gating does not apply. Furthermore, while electronic damage to crystalline samples occurs on similar time scales as in isolated molecules, atomic displacement is significantly reduced due to the confining crystal potential. In short, our results for radiation damage must not be transferred literally to the SFX case.

For each pulse, we carry out 25 MD simulations, giving a total of 1000 MD runs. The simulation time is fixed to three times the FWHM pulse duration. 100 snapshots of each trajectory, *i.e.* atom positions and electron-density distributions, are saved during each run.

At each time step during the simulation, we calculate the X-ray intensity scattered by the sample. The instantaneous scattering is given by the momentary distributions of electrons and X-ray pulse intensity. We then calculate a diffraction pattern by integrating the instantaneous scattering over the pulse duration and over the solid angle covered by each pixel. For a given electronic configuration, the time-integrated scattered intensity, including coherent (elastic) scattering from bound electrons and incoherent Compton scattering from bound and free electrons, reads 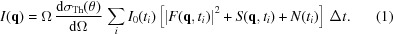
The wavevector **q** is determined by the distance of the assumed pixel area detector from the sample and the pixel coordinates in the detector plane, Ω is the solid angle spanned by the respective detector pixel, dσ_Th_/dΩ is the differential Thomson cross section, *I*_0_(*t*) is the FEL intensity as a function of time, which we take from the X-ray propagation results, *F*(**q**, *t*) is the bound-electron form factor for coherent scattering, *S*(**q**, *t*) and *N*(**q**, *t*) denote the incoherent contributions from bound and free electrons, respectively, *t_i_* is the time stamp of the *i*th snapshot, and Δ*t* is the time step of the simulation.

From each trajectory we calculate 200 diffraction patterns. Every pattern calculation starts from a random rigid rotation of the sample’s atom coordinates to simulate the erratic *a priori* unknown and uncontrolled orientation of sample molecules in the X-ray beam.

In our simulations, the detector is represented by a square pixel array (80 × 80 pixels) in a plane perpendicular to the beam axis located at a distance of 13 cm downstream from the sample. The pixel size is 1200 µm. Hence, one pixel of our simulated detector corresponds to a 6 × 6 pixel array in the AGIP detector (AGIPD) (Allahgholi *et al.*, 2015 ▸) planned for the SPB/SFX instrument (Mancuso *et al.*, 2013 ▸). These figures result in a half-period resolution of 3.6 Å at the detector edge.

The total scattered intensity at each pixel is divided by the central photon energy to yield a photon count *n*_0,*j*_, where *j* indexes the detector pixel. Poisson noise is added by drawing the detected photon count *n_j_* from a Poisson distribution 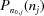 with *n*_0,*j*_ 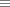 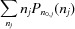.

### 2.4. Orientation   

Lastly, the simulated diffraction patterns are fed into the EMC algorithm to generate a three-dimensional diffraction volume. We calculate multiple such three-dimensional diffraction volumes (typically five), starting each EMC run from a different random initialization. If the input data had zero noise, *i.e.* if the differences between diffraction patterns originated only from different sample orientations, not from noise, each EMC run would yield the same three-dimensional output. Hence, a normalized root-mean-square (r.m.s.) variation of all EMC runs is a suitable figure of merit to measure the consistency of the oriented three-dimensional data and the likelihood that EMC finds the true orientation of noisy and shot-to-shot fluctuating individual two-dimensional patterns. The coefficient of variation σ_v_ as a function of resolution *q* = |**q**| was defined by Yoon *et al.* (2016 ▸) as 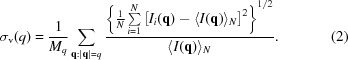
The inner sum is the mean-square deviation from their average over *N* orientation runs. The r.m.s., normalized to the average intensity, is then summed over all voxels within a resolution shell 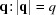 and divided by the number of voxels in the resolution shell *M_q_*. This metric uses the simulated data alone and no *a priori* information such as the original sample position. Hence it may be applied to experimental data, where the original structure and sample orientation are truly unknown, as opposed to *e.g.* the misorientation angle (Tegze & Bortel, 2012 ▸; Morawiec, 2004 ▸), which calculates the angular distance between the recovered orientation and the original sample orientation. An alternative metric not requiring the true orientation is the so-called correlation *C* factor (Tegze & Bortel, 2016 ▸).

## 3. Results and discussion   

**Figure 1:**
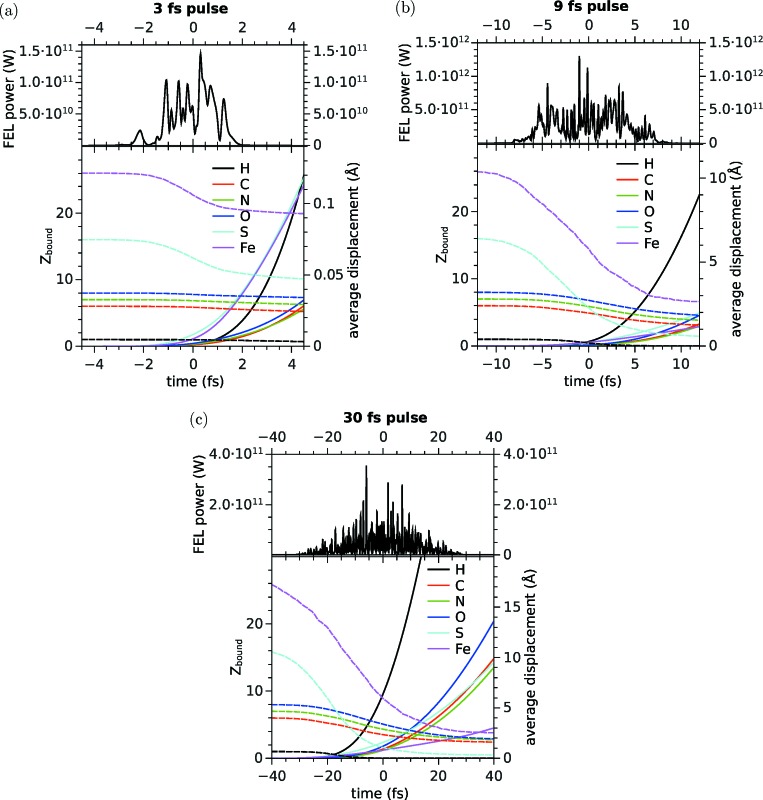
The temporal structure of the simulated X-ray pulse, the average number of bound electrons (*Z*_bound_, dashed curves) and the average atomic displacements (solid curves) in the 2nip sample as a function of time for pulse durations of (*a*) 3 fs, (*b*) 9 fs and (*c*) 30 fs.

Fig. 1 ▸ shows the temporal intensity variation of one representative X-ray pulse simulation from the output of the X-ray source simulation. Underneath, we show the evolution of the number of bound electrons (dashed curves) and atomic displacement from initial positions (solid curves) as a function of time. Both quantities are averaged over all atoms of a given species and over all sample trajectories. As expected, the electronic and ionic radiation damage becomes more severe if the pulse duration is increased. At the shortest pulse duration, the ionization stays below a level of 30%, even for the heaviest species S and Fe. The average displacement is below 0.1 Å over the entire duration of the pulse, which is negligible compared with the displacement of a few ångströms in the 9 fs pulse and that of 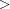
10 Å in the case of the 30 fs pulse.

**Figure 2:**
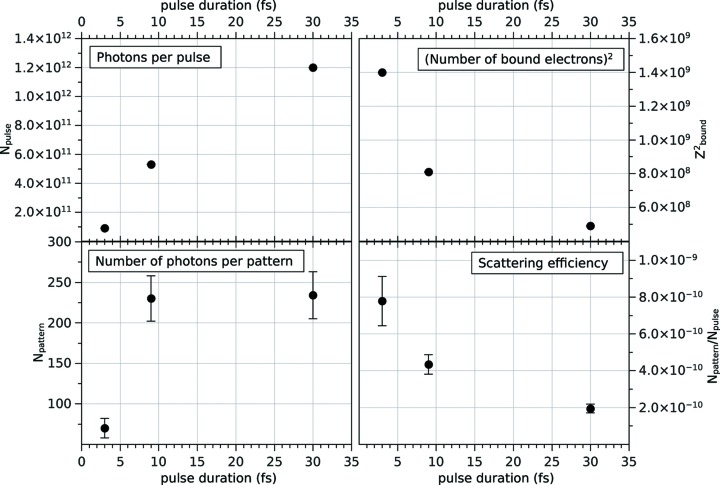
(Top left) The number of photons per pulse incident on the sample (*N*_ph,pulse_) as a function of pulse duration. (Bottom left) The number of detected photons per diffraction pattern (*N*_ph,det_). (Top right) The square of the average number of bound electrons in the sample molecule (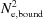) in the middle of the pulse. (Bottom right) The scattering efficiency *N*_ph,det_/*N*_ph,pulse_. The decrease in *N*_e,bound_ as a consequence of ionization processes results in a reduced scattering efficiency with increasing pulse duration. Nevertheless, the total number of detected photons increases, since the longer pulses contain more photons.

From the propagated pulse data, the radiation damage results and the diffraction patterns, we extract the average number of photons per pulse *N*_ph,pulse_ (top left in Fig. 2 ▸), the number of detected photons per simulated diffraction pattern *N*_ph,det_ = 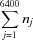 averaged over all simulated patterns (bottom left), the number of bound electrons in the sample *N*_e,bound_ at the middle of the pulse averaged over all simulated sample trajectories (top right), and the observed scattering efficiency, taken here as the ratio *N*_ph,det_/*N*_ph,pulse_ (bottom right).

For a pulse duration of 3 fs, *N*_ph,det_ is reduced by more than a factor of three compared with both the 9 fs and 30 fs cases. At 9 fs, *N*_ph,det_ is approximately equal to the 30 fs case because, coincidentally, the lower fluence in the 9 fs pulses is counterbalanced by an increase in scattering efficiency due to the lower degree of ionization.

**Figure 3:**
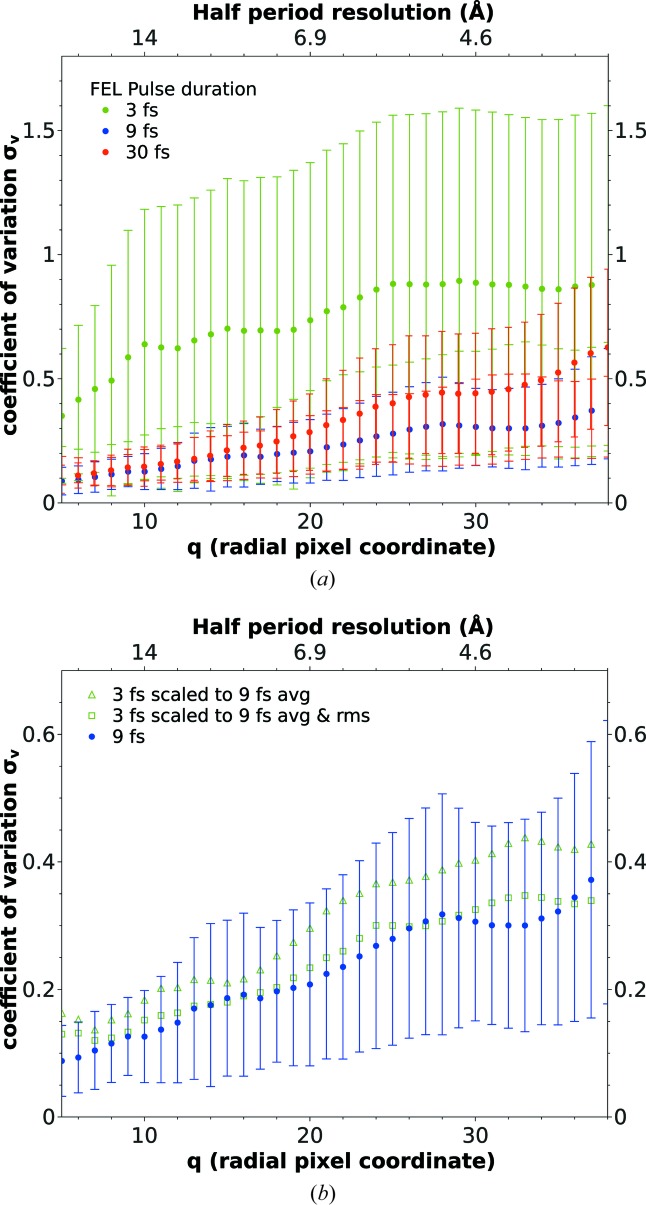
(*a*) The coefficient of variation of oriented three-dimensional diffraction volumes for pulse durations of 3, 9 and 30 fs. (*b*) The coefficient of variation for a pulse duration of 9 fs and re-scaled coefficients for 3 fs. Triangles: every pattern has been multiplied by a constant factor of 3.3 to match the average photon count in the 9 fs patterns. Squares: every 3 fs pattern has been multiplied by an individual factor such that the average and r.m.s. photon counts match the 9 fs data.

We now turn to the question of how the simulation results influence the consistency of oriented diffraction data measured by the coefficient of variation [equation (2)]. Fig. 3 ▸(*a*) shows σ_v_ as a function of the radial detector pixel coordinate (lower *x* axis) and as a function of the half-period resolution (upper *x* axis). The error bars represent the r.m.s. deviation over the resolution shell. Red and blue circles represent diffraction data from simulations using 30 and 9 fs pulses, respectively, taken from Yoon *et al.* (2016 ▸), and green circles correspond to the 3 fs simulations. The 30 and 9 fs diffraction data yield nearly identical variations down to resolutions of 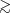
10 Å (14 pixels). For lower resolutions (larger pixel numbers), the 30 fs curve increases more quickly than the 9 fs curve. This 10 Å length scale agrees with the order of average atomic displacement found in the MD simulation of the sample towards the end of the 30 fs pulse (Fig. 1 ▸). The shorter 9 fs pulse clearly mitigates ionic damage since the coefficient of variation rises much more slowly as a function of radial pixel coordinate beyond 10 Å resolution. These findings also correspond with the improved electron-density reconstruction from the 9 fs data compared with the 30 fs data, as demonstrated by Yoon *et al.* (2016 ▸). While low-resolution features (*e.g.* the size and shape of the molecule) are recovered equally well in both cases, finer structures and the sample surface are resolved more markedly in the 9 fs reconstructions. This underlines the usefulness of the coefficient of variation as a measure of data quality as it indicates down to which length scales electron-density reconstructions are trustworthy. In our example, σ_v_ 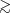 0.2 indicates a loss of accuracy at the 10 Å length scale. At this point we would like to remind the reader that our analysis applies to the case of SPI, whereas SFX is much less affected by radiation damage due to the self-gating effect (Barty *et al.*, 2011 ▸), as discussed above.

Analyzing the 3 fs curves in Fig. 3 ▸(*a*), we find that σ_v_ is more than a factor of two larger than in the 9 fs case over the entire range of *q* values and quickly approaches σ_v_ = 1, indicating that variations between individual orientation runs are of the same magnitude as the average. Also, the variation in a given resolution shell (vertical error bars) is nearly twice as large as in the 9 fs case. Despite the low ionization and negligible atomic displacement, the signal-to-noise level for the 3 fs diffraction data is insufficient for the orientation algorithm to work consistently and robustly.

Fig. 3 ▸(*b*) represents additional analysis. The green triangles mark the variation coefficient of five EMC runs, starting from the simulated 3 fs diffraction data after multiplying each pixel value by a factor 3.3, such that the resulting average total photon count equals the average for the 9 fs case. The green squares represent the orientations obtained after scaling the 3 fs data such that we obtain both the average photon count and the r.m.s. photon count of the 9 fs diffraction data. The resulting coefficient of variation agrees well with the 9 fs data (blue circles) within the error bars, while the green triangles lie systematically above the 9 fs data. This analysis shows that the quality of the 3 fs data cannot be improved by simply increasing the number of simulated diffraction patterns, since this would not reduce the signal-to-noise ratio.

These results make it clear that any electron-density reconstructions from our 3 fs data would be meaningless and we did not carry out this last step of the simulation pipeline.

## 4. Conclusions and outlook   

In conclusion, our realistic start-to-end simulations indicate that 3 fs pulses contain too few photons to allow consistent orientation of diffraction patterns from our sample protein 2nip. Over the range of resolutions studied here (∼3–15 Å), the optimal pulse duration for molecules of comparable size is closer to 9 fs than to 3 fs. Although shorter pulses mitigate electronic radiation damage (ionization), longer pulses are preferable as they contain five times more photons, which leads to much better photon statistics in the detected diffraction patterns and hence allows a more accurate reconstruction of the three-dimensional diffraction data.

Our results for the optimal pulse duration for single-particle imaging of a given sample and a given experimental configuration are, of course, strictly valid only for molecules of a size comparable with 2nip (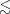
10 nm). Any conclusion regarding larger, more realistic, samples could only be speculative at this point. In particular, radiation damage time scales, such as the onset of electrostatic trapping and nano-plasma expansion, differ significantly for small and large samples (Hau-Riege *et al.*, 2004 ▸). The availability of simulation data for only one small molecular sample does not justify their extrapolation to larger particles, as strong nonlinearities can be expected at such scaling.

Simulations for larger particles and various photon energies are planned as the next step towards a simulation database which will also allow the development and tuning of analytical models, *e.g.* for radiation damage time scales (Hau-Riege *et al.*, 2004 ▸), and the inference of the minimum number of detected photons needed for the reconstruction of a molecule of a certain size and at a targeted resolution. Such an expression will help to define the requirements for experimental parameters (pulse duration and fluence) for the measurement of unknown structures. These future simulations of larger particles should then also include the variation in X-ray intensity across the sample and a realistic simulation of the detector response using the tools described by Joy *et al.* (2015 ▸) and Rüter *et al.* (2016 ▸).

Our simulation pipeline is organized in a way that facilitates the usage of different methods and algorithms or different implementations of a given algorithm, and thereby enables a comparison of the effect of such different tools on the simulated diffraction pattern and their impact on the orientation and density reconstruction for a given fixed set of X-ray pulse parameters, sample molecule and detector geometry. Such a comparison is, however, not within the scope of the present paper, and we remark here only briefly on the possible impact of different radiation damage models and orientation algorithms.

As shown by Moribayashi (2010 ▸), electron-distribution functions modelled with a continuum approach and MD simulation results are in good agreement for close-to-spherical samples ∼10 nm in diameter. The sample molecule used in this work is of similar size and hence we would not expect any major differences if a continuum model were used. On the other hand, continuum models, as mentioned above, do not allow us to achieve the same atomic resolution in the simulation as atomistic MD simulations do. Also, if kept on the level of single-particle density, continuum models do not reproduce interparticle correlations and therefore require corresponding corrections.

Regarding alternative orientation algorithms, it should be noted that the aforementioned common-arc method is more sensitive to noise in the diffraction pattern and relies on amplification of the signal-to-noise ratio by averaging over patterns of similar orientation which have to be classified by means of correlation analysis. The latter makes this approach less favourable for analysing large data sets of the order of 10^5^ to 10^6^ diffraction patterns due to the 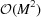 growth in CPU time and memory requirements, so we discard it as an alternative. Another algorithm presented by Tegze & Bortel (2012 ▸) overcomes this limitation and also shows better scaling compared with EMC in the dimensionless parameter *R* = *D*/*d*, where *D* is the diameter of the sample and *d* is the desired spatial resolution [*R* ^5^log*R* *versus* *R* ^6^ − *R* ^8^ (Moths & Ourmazd, 2011 ▸)]. A comparison of this algorithm with EMC, using noisy diffraction data, is, to the best of our knowledge, outstanding and would make an important contribution to the field.
